# Heart Rate Variability Parameters During Psychogenic Non-epileptic Seizures: Comparison Between Patients With Pure PNES and Comorbid Epilepsy

**DOI:** 10.3389/fneur.2020.00713

**Published:** 2020-08-07

**Authors:** Andrea Romigi, Giada Ricciardo Rizzo, Francesca Izzi, Maria Guerrisi, Marco Caccamo, Federica Testa, Diego Centonze, Nicola B. Mercuri, Nicola Toschi

**Affiliations:** ^1^IRCCS Neuromed Sleep Medicine Centre, Pozzilli, Italy; ^2^Neurophysiopathology Unit, Department of Systems Medicine, Sleep Medicine Centre, Tor Vergata University and Hospital, Rome, Italy; ^3^Medical Physics Section, Department of Biomedicine and Prevention, University of Rome “Tor Vergata”, Rome, Italy; ^4^Department of Neuroscience, “Tor Vergata” University, Rome, Italy; ^5^Department of Radiology, Athinoula A. Martinos Center for Biomedical Imaging, Boston, MA, United States; ^6^Harvard Medical School, Boston, MA, United States

**Keywords:** heart rate variability, PNES, autonomic nervous system, comorbid epilepsy, videoEEG

## Abstract

**Introduction:** Psychogenic non-epileptic seizures (PNES) may resemble epileptic seizures. There are few data about ictal ANS activity alterations induced by PNES in patients with pure PNES (pPNES) compared to PNES with comorbid epilepsy (PNES/ES). We aimed to compare heart rate variability (HRV) parameters and hence autonomic regulation in PNES in epileptic and non-epileptic patients.

**Methods**: We obtained HRV data from video-electroencephalography recordings in 22 patients presenting PNES (11 pPNES and 11 PNES/ES) in awake, and supine states. We calculated HRV parameters in both time and frequency domains including low frequency (LF) power, high frequency power (HF), LF/HF ratio, square root of the mean of the sum of the squares of differences between adjacent R wave intervals (RMSSD) and the standard deviation of all consecutive R wave intervals (SDNN). We also evaluated approximate entropy (ApEn), cardiosympathetic index (CSI), and cardiovagal index (CVI). Four conditions were considered: basal condition (BAS), before PNES (PRE), during PNES (ICT) and after PNES (POST).

**Results:** HRV analysis showed significantly higher ICT LF and LF/HF ratio vs. each condition. We also found higher POST HF vs. PRE and BAS, lower RRI in ICT vs. each condition and PRE vs. BAS. POST RMSSD was significantly higher compared to all other states. ICT CSI was significantly higher compared to all other states, whereas CSI was significantly lower in POST vs. PRE and PRE CVI lower than ICT and higher in POST vs. BAS and PRE. Also, ICT ApEn was lower than in all other states. Higher LF in pPNES vs. PNES/ES was also evident when compared across groups.

**Significance:** A few studies examined HRV alterations in PNES, reporting high sympathetic tone (although less evident than in epileptic seizures). Our data suggest a sympathetic overdrive before and during PNES followed by a post-PNES increase in vagal tone. A sympathovagal imbalance was more evident in pPNES as compared to PNES/ES.

## Introduction

The Diagnostic and Statistical Manual of Mental Disorders (DSM V) defines psychogenic non-epileptic seizures (PNES) as a conversion disorder (functional neurological symptom disorder) characterized by attacks or seizures (1). A diagnosis of PNES should demonstrate incompatibility between such episodes and neurological or medical disorders as well as exclude the possibility that PNES may be better be explained by medical or mental disorders (2, 3). PNES may resemble epileptic seizures, even if are not associated with epileptiform discharges (3). Nevertheless, a variable share of patients with PNES show comorbid epilepsy (4–7). The pooled frequency of epilepsy among patients with PNES is ~22% (7). Several studies demonstrated a high rate of psychiatric disorders in patients affected by pure PNES (pPNES), and similarly a high frequency of anxiety disorders, affective disorders, and other psychiatric disorders was also described in patients with PNES with comorbid epilepsy (PNES/ES) (4, 6, 8, 9).

While several studies have investigated interictal autonomic nervous system (ANS) changes in epilepsy (10–21) and PNES (22–26), no information is yet available about putative differences in ANS activity alterations induced by pPNES as compared to PNES/ES. In this context, a sympathetic overdrive higher than that observed during PNES was described in temporal lobe seizures (24, 27). Heart rate (HR) changes with respect to baseline were significantly higher during epileptic seizures than during PNES (28). On the other hand, Reinsberger et al. could not find any difference in HR changes between non-convulsive seizures and non-convulsive PNES (23). Accordingly, it was recently suggested that more advanced analysis of heart beat time series, such as heart rate variability (HRV) analysis, may be able to distinguish the ANS-related effects of PNES and epileptic seizures (25).

HRV analysis is an easy-to-use tool able to non-invasively to assess autonomic abnormalities (29). It has been able to demonstrate sudden and markedly higher sympathetic activity before and during seizures in a number of studies on epileptic patients (20, 21, 30). Several authors hypothesized that this finding may represent a direct link between the epileptic focus and the cortical areas connected to the ANS (10, 31). Interesting, similar findings were described in PNES (25–27). Jeppesen et al. (27) found higher maximum sympathetic activity (as estimated by the cardiosympathetic index—CSI) during epileptic seizures as compared to during PNES. Still, the authors highlighted notable variability in HRV parameters in both groups, rendering the use of ANS data to distinguish epileptic seizures from PNES arduous. On the other hand, Ponnusamy et al. (25) reported higher sympathetic and lower parasympathetic tones during epileptic seizures as compared to during PNES. However, in this paper HRV analysis was performed by averaging the whole seizure period for both epileptic seizures and for PNES. Importantly, the different duration of ictal episodes (both epileptic seizures and PNES) can bias short time HRV-analysis (25, 29). Similarly, Van del Krujis described increased sympathetic tone before PNES episodes followed by a rise in vagal tone during and after PNES (26). Also, little is known about HRV changes in pPNES and PNES/ES. The goal of our study was to assess the effects of PNES of ANS activity in these two groups evaluating different states (basal condition, before PNES, during and after PNES) in order to distinguish possible different ANS profiles as a function of state and group and, in particular, to test the hypothesis of a more pronounced HRV changes in patients with PNES/ES as compared to pPNES.

## Methods

### Subjects

Patients with PNES were retrospectively recruited from the Outpatients Epilepsy Center at the University of Rome Tor Vergata General Hospital from 2010 to 2014 after receiving a diagnosis by two experienced neurologists (AR, FI) as well as by a neurophysiological trainee (GRR). We defined the diagnosis of definite PNES in patients showing spontaneous or provoked seizures recorded with video-EEG (32). All episodes lacking ictal EEG were considered consistent with the habitual witnessed seizures. Included criteria for this retrospective cohort study were patients who were at least 18 years old.

Group assignment was conducted as follows: each patient was classified as affected by pPNES when two concomitant conditions were met: (1) video-EEG-documented PNES (2) lack of ictal EEG epileptiform activity. Patients were classified as affected by PNES and comorbid epilepsy when the following conditions were met (1) video EEG-documented PNES; (2) a confirmed diagnosis of epilepsy before PNES appearance (3) epileptic seizures with prototypical semeiology that could be differentiated from PNES and (4) interictal or ictal epileptiform discharges during typical epileptic seizures. In all epileptic patients, the onset of epilepsy was documented to have occurred before the onset of PNES. In addition, patients whose PNES due to hyperventilation maneuvers were excluded from the analysis, as this would affect HRV analysis.

This study was approved by the Institutional Review Board (IRB) of the University of Rome Tor Vergata. Given that this was a retrospective study, the IRB specifically waived the necessity for participant consent. Data were anonymized by removal of direct identifiers from the data file and also de-identified before analysis.

Exclusion criteria were (1) intake of drugs interfering with ANS function other than antiepileptic drugs (AEDs), (2) history of heart failure, endocrine and metabolic disorders, uremia or any other condition involving ANS. All video-EEG recordings were evaluated by two independent epileptologists (AR, FI) as well as by a neurophysiological trainee (GRR). Awake episodes occurring in supine state and at rest with artifact-free preictal, ictal, and postictal ECG were selected. The onset and the end of PNES were defined by typical behavioral changes and clinical manifestations of PNES with or without apparent alteration of consciousness.

We defined four conditions in a consensus-based manner:

1. Basal condition (BAS): 2-min of artifact-free resting ECG obtained before PNES and during wakefulness (acquired between 9 and 11 a.m. in order to minimize circadian HRV variations);
2. Preictal (PRE): 2-min of artifact-free ECG immediately before PNES onset
3. Ictal (ICT): 2-min of artifact-free ECG immediately after PNES onset and comprising the entire seizure duration
4. Post-ictal: (POST) 2-min of artifact-freeECG immediately after seizure end.

### ECG Samples and RR Series Construction

We carried out bipolar ECG recording from lead I of a 12-lead ECG by the ECG channel of the EBNNeuro EEGNet System (EBNNeuro – Florence Italy). The sampling rate of ECG data was 256 Hz. ECG was exported from the EEG system in the European Data Format. We processed ECG data with custom-built code in Labview 2013^©^ and Mathematica 12^©^. QRS complex recognition and R wave detection were performed by a multiscale wavelet-based peak detection algorithm, construction of an RR interval (RRI) time series by calculating the delay between consecutive R-peaks, and obtaining a resampling of the RRI series at a frequency of 8 Hz using cubic splines as basic functions. Visual editing before interpolation and resampling of all RRI series was achieved in combination with the native ECG trace to remove erroneously recognized R waves and to perform insertion of missed R beats. Moreover, interpolation of adjacent R waves after elimination of ectopic heartbeats was performed as recommended elsewhere (33). We employed the same methodology described in our previous study on temporal lobe epilepsy (21).

### HRV Analysis

The HRV analysis methodology employed in this paper has been reported elsewhere (21). To forego the statement of stationarity which is mandatory for conventional, Fourier-Transform based methods to be applicable, we analyzed the interpolated and resampled RRI by a time-frequency decomposition technique. We used the Hilbert transformation to make the signal analytic and we acquired a time-frequency representation of the signal for each RRI sample by calculating its Smoothed Pseudo Wigner Ville distribution (time smoothing window: Hamming window of 42 samples, frequency smoothing window: Hamming window of 129 samples) (21, 34). We entered a total ECG length of 2 min for each series (45 s before and 45 s after the condition to evaluate) into the analysis. Afterward, the time-frequency representation was constrained to the 30 s under investigation, after which we estimated average Low Frequency Power (LF, 0.04–0.15 Hz) and High Frequency Power (HF, 0.15–0.4 Hz) by time-averaging for each patient and each state. Whereas, LF power represents the combined modulation of the sympathetic and the parasympathetic tones, HF mostly is the expression of vagal activity (35). The LF/HF ratio is a measure of the balance between both branches of ANS. We did not evaluate the Very Low Frequency (VLF, ≤ 0.04 Hz) component due to the necessity of long-term ECG recordings. We also studied two time-domain HRV parameters: SDNN (Standard deviation of all NN intervals) and RMSSD (root mean square of the difference of adjacent NN intervals). SDNN shows an overall estimate of HRV and gives information concerning all its components. On the other hand, RMSSD is a powerful parameter to estimate parasympathetic activity in short-term recordings (33). Since SDNN depends on record duration (33), it should be compared between recordings of similar length. In addition, we considered two variables derived from the Poincarè Plot of the RRI series (where each RRI is plotted against the following one): the cardiovagal index (CVI = Log10 [L ·T]) and cardiosympathetic index (CSI = L/T) (21, 25, 36). The transverse axis (T) in the Poincarè Plot represents the beat-to-beat variability, with deviations along this axis predominantly due to vagal effect. The longitudinal axis (L) reflects the overall range of RRIs due to both and branches (21, 25). It seems that these parameters provide complementary evidence about parasympathetic and sympathetic involvement in HRV when compared to data obtained only by spectral analysis. Lastly, we analyzed the approximate entropy (ApEn), which reveals changes not evident to visual inspection. ApEn is used to quantify regularity vs. randomness, the consistency of variations in a time series (as the RRI time series) (21, 25). HRV parameters and their pathophysiological significances are listed in Table 1.

**Table 1:** Overview of HRV parameters utilized in the study.

	**Variable**	**Unit**	**Definition**	**Interpretation**	**Frequency range**
Time domain	RRI	s	Cardiac beat-to-beat interval	Measure of physiological phenomenon	NA
	SDNN	ms	Standard deviation of all NN intervals	Total HRV	NA
	RMSSD	ms	The square root of the mean of the sum of the squares	Parasympathetic response	NA
	CSI		Cardiac Sympathetic Index (Poincarè Plot)	Sympathetic response	NA
	CVI		Cardiac Vagal Index (Poincarè Plot)	Parasympathetic response	NA
	ApEn		Approximate Entropy	Measure of regularity vs. randomness and quantifies the predictability of fluctuations in a time series (i.e., RRI).	NA
Frequency domain	LF	s^2^	Power in low frequency range	Mix between the sympathetic and vagal influences	0.04–0.15 Hz
	HF	s^2^	Power in high frequency range	Parasympathetic response	0.15–0.4 Hz
	LF/HF	–	Ratio	Relative balance of sympathetic to parasympathetic responses	NA

### Statistical Analysis

*T*-test and chi square tests were employed to compare continuous and dichotomous demographic variables respectively. *p*-values below 0.05 were considered significant. We confirmed HRV parameters normality by the Mardia Coefficient of Multivariate Kurtosis, after which we compared all HRV data across groups and conditions using a General Linear Model (GLM) which modeled subject as a “between-subject” factor (pPNES and PNES/ES) and one 4-level “within-subject” factor (“state”: BAS, PRE, ICT, POS). When the overall effect of a factor was seen to be statistically significant (*p* < 0.05), we employed Fisher LSD was performed as a *post-hoc* test. Besides, the GLM considered age and gender as nuisance covariates. A *p*-value < 0.05 was considered statistically significant. All statistical analysis was carried out in the Statistica® 10.0 software package (Statsoft, USA).

## Results

### Subjects

**Figure 1:**
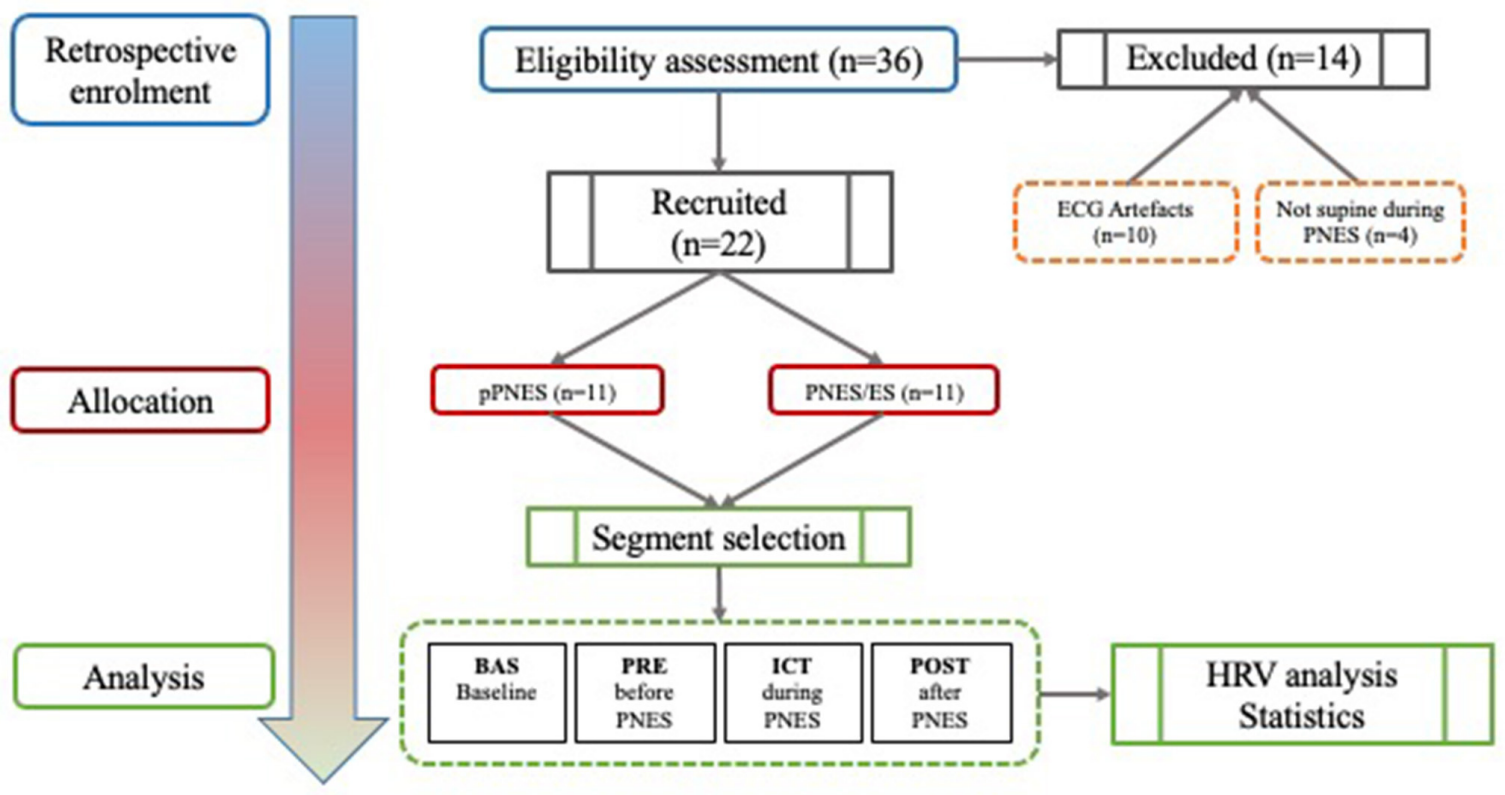
Flow diagram of the study.

Twenty-two patients out of a total of 36 (61%) subjects were selected for the study (2 males, 20 females, mean age 34 ± 14.5). Out of the initial 36, 14 patients were excluded: *n* = 3 patients suffered a PNES while not in a supine position (hence possibly interfering with HRV analysis), and *n* = 11 patients had ECG artifacts in one or more conditions (BAS/PRE/ICT/POST) that would have affected HRV analysis. Out of the remaining 22 patients, eleven patients were affected by pPNES (mean age 26 ± 10.5, 9 F, 2 M) and 11 patients were affected by PNES and comorbid epilepsy (mean age 41 ± 14.8, 11 F). Nine patients were affected by focal epilepsy (6 patients with temporal lobe epilepsy, 3 patients with extratemporal origin epilepsy) and 2 patients were affected by idiopathic generalized epilepsy (1 patient affected by Juvenile Myoclonic Epilepsy, and 1 patient affected by epilepsy with generalized tonic-clonic seizures alone). All epileptic patients were treated with AEDs (5/11 monotherapy and 6/11 polytherapy) whereas 6 out 11 (36%) of patients with pPNES were treated with AEDs (5 monotherapy and 1 bi-therapy), 2 patients received valproate as mood stabilizer; interictal epileptiform discharges were evident in 10 out 11 patients with PNES/ES vs. 2/11 (18%). The first observable clinical manifestation was defined as the onset of PNES. No more than one PNES for each patient was evaluated. Figure 1 depicts our study workflow. Clinical and demographic variables are reported in Table 2. Furthermore, all patients were right-handed. We found a significantly higher age (*p* = 0.01) and age at PNES onset (*p* = 0.02) in PNES/ES. Interictal EEG abnormalities (91 vs. 18% *p* = 0.0019), the number of patients treated with AEDs (100 vs. 54.5% *p* = 0.03) and the median number of AEDs (*p* = 0.001) was significant higher in PNES/ES. Psychiatric disorders were more prevalent in pPNES than PNES/ES (100 vs. 18% *p* = 0.0002). Psychiatric diagnoses in pPNES were conversion disorders (2/11), post-traumatic stress disorder (3/11), affective disorders (3/11), and anxiety (1/11), whereas in PNES/ES we affective disorders in both patients. Due to the retrospective design of our study, the precise magnitude of psychiatric disorders may be underestimated in PNES/ES group. No differences in terms of for sex, family history of epilepsy and psychiatric disorders, PNES duration and motor symptoms were found between groups.

**Table 2:** Demographic and clinical features of the pure PNES and PNES with comorbid epilepsy groups.

	**pPNES (*n* = 11)**	**PNES/ES (*n* = 11)**	***p***
(Male/Female)	2/9	0/11	n.s.
Age (mean ± SD)	26 ± 10.5	41 ± 14.8	0.01^*^
Age of epilepsy onset (mean ± SD)	–	19 ± 17.7	–
Age of PNES onset (mean ± SD)	24 ± 10.3	38 ± 16	0.02^*^
Disease (PNES) duration y (mean ± SD)	3.18 ± 2.13	3.45 ± 5.57	n.s.
Number of pts with AEDs (mean ± SD)	6/11 (54.5%)	11/11 (100%)	0.03^*^
Median number of AEDs (mean ± SD)	1 ± 0.67	2 ± 1	0.001^*^
Psychiatric disorders	11/11 (100%)	2/11 (18%)	0.0002^*^
Interictal paroxysmal EEG	2/11 (18%)	10/11 (91%)	0.0019^*^
Familial history of epilepsy	2/11 (18%)	2/11 (18%)	n.s.
Familial history of psychiatric disorders	1/11 (9%)	0/11 (0%)	n.s.
PNES duration min (mean±SD)	9.36 ± 9.15	11.7± 7	n.s.
Hypermotor PNES with counsciusness impairment	4/11 (36%)	3/11 (27%)	n.s.
Hypermotor PNES without counsciusness impairment	3/11 (27%)	4/11(36%)	n.s.
Hypomotor PNES with counsciusness impairment	2/11 (18%)	0/11 (0%)	n.s.
Hypomotor PNES without counsciusness impairment	2/11 (18%)	4/11(36%)	n.s.

* *Stastically significant (p < 0.05). pPNES, pure PNES; PNES/ES PNES with comorbid epilepsy*.

### Effect of “State”

The “state” factor was seen to be associated to a statistically significant effect (*p* < 0.01) in for all indices investigated. The results of *post-hoc* testing were as follows. Across all PNES (*n* = 22), LF was higher in ICT as compared to all states (BAS *p* = 0.04, PRE *p* = 0.0004 POST *p* = 0.005). LF/HF ratio was significantly higher in ICT vs. all conditions (BAS *p* = 0.04, PRE *p* = 0.04, POST *p* = 0.005). Likewise, lower RRI was evident in ICT as compared to all states (BAS *p* < 0.0001, PRE *p* = 0.0001, POST *p* = 0.00001), in PRE compared to BAS (*p* = 0.0001) and in POST vs. BAS (*p* = 0.001).

HF was significantly lower in PRE (*p* = 0.01) and in ICT (*p* = 0.007) vs POST. SDNN was significantly greater in PRE (*p* = 0.002), ICT (< 0.0001) and POST (*p* = 0.00003) states compared with BAS, and in ICT vs. POST (*p* = 0.0002). RMSSD was significantly higher in POST compared to BAS (*p* = 0.04), PRE (*p* = 0.007), and ICT (*p* = 0.2). Moreover, CSI was significantly higher in PRE vs. BAS (*p* = 0.0002) and vs. POST (*p* = 0.01) and ICT vs. BAS (*p* < 0.0001), and PRE (*p* = 0.02) and POST (*p* = 0.00001) states. On the other hand, CVI was significantly higher in POST vs. BAS (*p* = 0.00009) and vs. PRE (*p* = 0.003) and in ICT vs. BAS (*p* = 0.005). In addition, ApEn was lower in ICT vs. all conditions (BAS *p* ≤ 0.0001; PRE *p* = 0.009; POST *p* = 0.004); in PRE vs. BAS (*p* = 0.00008), in POST vs. BAS (*p* = 0.002). These data are summarized in Table 3.

**Table 3:** Differences in HRV metrics during each condition.

	**BAS Mean ± SD**	**PRE Mean ± SD**	**ICT Mean ± SD**	**POST Mean ± SD**	***P***
LF	13.05 ± 4.9	10.79 ± 3.42	15.75 ± 7.78*^#^*^,^^*^^,^^ç^	11.9 ± 3.49	^#^0.04; ^*^0.0004;^ç^0.005
HF	5.71 ± 1.93	4.79 ± 1.46	4.66 ± 2.26	6.19 ± 3.04**^§^**^,^^ç^	^§^0.01;^ç^0.007
LF/HF ratio	2.4 ± 0.69	2.4 ± 0.71	3.41 ± 2.88*^#^*^,^^*^^,^ ^ç^	2.02 ± 0.82	^#^0.04; ^*^0.04;^ç^0.005
RR interval	0.87 ± 0.07	0.81 ± 0.08^†^	0.74 ± 0.08*^#^*^,^^*^^,^ ^ç^	0.82 ± 0.05	†0.0001; ^#^ < 0.0001; ^°^0.001; ^*^0.0001;^ç^0.00001
RMSSD	0.2 ± 0.07	0.19 ± 0.04	0.20 ± 0.07	0.24 ± 0.09**^°^^,^^§^^,^^ç^**	^°^0.04; ^§^0.007;^ç^0.02
SDNN	0.04 ± 0.01^†^^,^ **^#^^,^** ^°^	0.05 ± 0.009^*^	0.07 ± 0.02	0.06 ± 0.01	^†^0.002; ^#^ < 0.0001; ^°^0.00003; ^*^0.0002
CSI	10.06 ± 1.65	13.78 ± 2.87	15.99 ± 4.97^*^^,^^#^^,^^ç^	11.43 ± 2.33	^†^0.0002; ^#^ < 0.0001; ^*^0.02; ^§^0.01^ç^0.00001
CVI	3.69 ± 0.27***^#^*^,^^°^**	3.75 ± 0.15**^§^**	3.86 ± 0.26	3.94 ± 0.28	^#^0.005;^°^0.00009; ^^§^^0.003
ApEn	0.58 ± 0.04	0.48 ± 0.06^†^	0.42 ± 0.10^*^^,^ ^#^^,^^ç^	0.5 ± 0.08^°^	^†^0.00008; ^#^ < 0.0001; ^°^0.002; ^*^0.009;^ç^0.004

*Across all PNES, LF, LF/HF ratio were higher in ICT as compared to all states; RRI lower in ICT vs. all states) in PRE vs. BAS and in POST vs. BAS (p = 0.001); HF significantly lower in PRE and in ICT vs. POST; SDNN significantly greater in PRE, ICT, and POST vs. BAS, and in ICT vs. POST; RMSSD higher in POST vs. BAS, PRE, and ICT; CSI higher in PRE vs. BAS and vs. POST and in ICT all states; CVI higher in POST vs. BAS and PRE and in ICT vs. BAS. Finally, ApEn lower in ICT vs. all conditions*.

*BAS, basal; PRE pre PNES; ICT PNES; POST dopo PNES; Level of significance P < 0.05*.

† *BAS vs. PRE*;

# *BAS vs. ICT*;

° *BAS vs. POST*;

* *PRE vs. ICT*;

§ *PRE vs. POST*;

ç *ICT vs. POST*.

### Effect of “Group”

When inspecting the effect of the “group” factor (pPNES vs. PNES/ES) irrespective of condition we found a significant higher LF value in patients with pPNES compared to patients with PNES/ES (*p* = 0.00012) (see Table 4). No further statistically significant differences in HRV parameters were seen across groups.

**Table 4:** Differences in HRV metrics between groups.

**HRV metrics**	**pPNES**	**PNES/ES**	***p***
LF	15.35 ± 6.05	10.39 ± 3.32	0.00012^*^
HF	5.40 ± 1.90	5.27 ± 2.67	N.S.
LF/HF	2.76 ± 2.15	2.35 ± 0.85	N.S.
RRI	0.82 ± 0.10	0.80 ± 0.09	N.S.
CSI	12.29 ± 4.24	13.34 ± 3.48	N.S.
CVI	3.83 ± 0.24	3.78 ± 0.29	N.S.
RMSSD	0.22 ± 0.07	0.19 ± 0.08	N.S.
SDNN	0.05 ± 0.02	0.05 ± 0.02	N.S.
ApEn	0.49 ± 0.11	0.49 ± 0.09	N.S.

*pPNES, pure PNES; PNES/ES PNES with comorbid epilepsy; Level of significance P < 0.05*.

* *Stastically significant (p < 0.05)*.

## Discussion

To our knowledge, our study is the first investigation to focus on HRV changes in pPNES compared with PNES/ES to test the hypothesis of different ictal HRV profile in patients with PNES/ES as compared to pPNES. In terms of demographics, while our study was not poised to determine demographic differences in PNES, our relatively small cohort confirmed that pPNES are mostly described in young adulthood during the second to fourth decades (5). Accordingly, our pPNES patients are younger than PNES/ES in our small sample. While previous studies have reported different demographics (37, 38), it should be noted that or patient cohort was 90% female. In this context, younger PNES patients have been seen to be mainly women (39, 40), with F/M ratios ranging up to 4.4 (40, 41). However, given that PNES diagnosis is more challenging in men than women even when employing video- EEG recordings (42), this ratio may be overestimated. Also, in our sample the age of PNES onset was higher in PNES/ES patients as compared to pPNES patients. Although no significant differences were found for age, age at onset, disease duration in a recent metanalysis comparing PNES and PNES/ES (7), we hypothesize that our narrow sample and long disease duration of epilepsy in PNES/ES may explain this difference. In addition, as reported elsewhere, we found higher psychiatric disorders, lower median number of AEDs and lower interictal discharges in pPNES (4, 7).

### Effects of PNES on HRV Parameters

HRV analysis across all groups demonstrated an ictal increase of sympathetic tone as showed by higher ICT LF as compared to BAS, PRE, and POST, higher ICT LF/HF ratio vs. BAS, PRE, and POST, and lower ICT RRI compared to BAS, PRE, and POST and PRE RRI compared to BAS and POST RRI vs. BAS. In addition, vagal tone (as estimated by HF) was seen to be lower in PRE and ICT as compared to post POST. The decrease of vagal tone during PNES was also confirmed by the significantly lower RMSSD value in BAS, PRE, and ICT vs. POST. Also, a significant increase of SDNN in PRE, ICT, and POST vs. BAS confirmed a peri-ictal HRV change during PNES. Moreover, CSI was significantly increased in PRE vs. BAS, in PRE vs. POST, in ICT vs. BAS, PRE, and POST states, confirming the ictal and preictal sympathetic activation. On the other hand, CVI was significantly increased in POST vs. BAS and vs. PRE, and in ICT vs. BAS, possibly evidencing a vasovagal involvement.

Our findings support the presence of a sympathovagal imbalance due to PNES, as demonstrated by changes in LF, HF, RRI, LF/HF ratio, CVI, CSI, RMSSD, and ApEn. These findings are particularly interesting because they reveal a combined activation of the sympathetic nervous system with a parallel reduction of vagal tone which is visible in both PRE and ICT conditions. This suggests that inputs from cortical or subcortical areas involved in and/or affected by PNES may explain the sympathovagal imbalance. Several previous studies have focused on interactions between PNES and ANS. Ponnusamy (25) found a significantly higher sympathetic tone and lower vagal tone in PNES less evident than during epileptic seizures. However, the authors employed average HRV measures across the entire episode duration for both PNES and epileptic seizures, without controlling for differences in seizure length—a factor which can introduce bias in HRV analysis especially in short time series (25, 29). Likewise, increased sympathetic tone before PNES episodes followed by an increase in vagal tone during and after PNES has been previously described (26). Also, Reinsberger (24) determined ictal sympathetic changes both in epileptic patients and PNES patients by electrodermal activity (EDA). Still, EDA alterations during PNES exhibited high variability. In our relatively small sample, we confirmed the sympathovagal alteration before, during and after PNES through established HRV analysis tools suitable for short-term recordings (20, 21). In addition, we found significantly lower ApEn in ICT vs. each condition (BAS; PRE; POST); in PRE vs. BAS, in POST vs. BAS. ApEn measures changes in heart rate dynamics which are not visually detectable, by quantifying the degree of regularity or predictability of a time-series. Higher values of ApEn are often found in healthy conditions, whereas a pathological status is often associated with lower ApEn also in psychiatric disorders (43). In this study, ApEn estimates were employed in conjunction with time and frequency-domain HRV analysis, and a joint interpretation allows to speculate that the lower ApEn during PNES confirms the predominance of the sympathetic branch of ANS.

### Effects of Group

To-date, no data are available regarding differences in ANS metrics between PNES and PNES/ES. Higher LF in pPNES may suggest an alteration of the adaptive cardiac reaction to stressful situations or unexpected cardiovascular difficulties, also recognized as “*cardiac resilience*” (21, 44–46). Therefore, our data are consistent with a sympathetic and parasympathetic imbalance and may show a tendency to a “pro-arrhythmic” disorder in pPNES.

Although the cortical control of ictal cardiovascular response seems to be influenced by a lateralization of ANS regulation where the pharmacologic inactivation of the right hemisphere or electrical stimulation of the left side leads predominately to a cardiodepressive response, whereas left-sided inactivation or right-sided stimulation results in an increased heart rate (47, 48), we cannot evaluate effects of localization and lateralization of epilepsy in our small sample. However, our finding is interesting because it aligns with and adds to recent finding in sudden unexpected death in epilepsy (SUDEP) (49). Verducci et al. identified 13 patients with PNES/ES and SUDEP (49). These authors showed that compared to SUDEP cases in patients affected only by epilepsy, those with PNES/ES were younger at the time of death and the mean delay between PNES recognition and SUDEP was merely 3 years. They hypothesized that for some patients with epilepsy, the presence of significant life stressors (i.e., physical, sexual and psychological abuse, serious economic, or relationship issues) may explain the appearance of PNES but may also increase risk of SUDEP. Therefore, PNES may increase the risk of SUDEP when compared with ES with similar demographic features (age of epilepsy onset, rates of neuropsychiatric comorbidity, age of death, AEDs adherence). In this context, sympathetic overdrive in pPNES may confirm this arrhythmogenic predisposition in PNES which, given the above mentioned association of PNES with higher mortality (50) and SUDEP (49), should be addressed promptly by specialists.

## Conclusion

Our study is affected by some limitations. These include the use of a retrospective design, the small sample size, the heterogeneity of diagnosis, localization, and lateralization of epilepsy in PNES/ES and of ictal semeiology of PNES in both groups. However, our data support the hypothesis of an early involvement of the ANS in PNES patients, which is more evident in subjects with pPNES. Although several attempts failed to identify ANS biomarkers to distinguish PNES and epileptic seizures (22, 24–27, 51), our data seems to confirm sympathovagal imbalance during PNES. LF alteration reflects both a sympathetic and parasympathetic tone modulation and baroreflex sensitivity (52, 53) more evident in pPNES than PNES/ES. The sympathetic overdrive in PNES may represent an electrophysiological marker and the pathophysiological basis of a probable higher mortality risk in these patients (49, 50). Studies in larger samples may be of aid in distinguishing ANS patterns specific to PNES subtypes as well as to different comorbid epileptic syndromes.

## Data Availability Statement

The raw data supporting the conclusions of this article will be made available by the authors, without undue reservation.

## Ethics Statement

The studies involving human participants were reviewed and approved by Institutional Review Board (IRB) of the University of Rome Tor Vergata. Written informed consent for participation was not required for this study in accordance with the national legislation and the institutional requirements.

## Author Contributions

AR and NT design and conceptualized study, analyzed the data, and drafted the manuscript for intellectual content. GR analyzed the data and drafted the manuscript for intellectual content. FI analyzed the data. MG, MC, FT, DC, and NM drafted the manuscript for intellectual content. NT Design and conceptualized study, analyzed the data. All authors contributed to the article and approved the submitted version.

## Conflict of Interest

The authors declare that the research was conducted in the absence of any commercial or financial relationships that could be construed as a potential conflict of interest.
